# On the Physiological and Psychological Phenomena of Dreams and Apparitions

**Published:** 1857-04-01

**Authors:** 


### Art. V.—ON THE PHYSIOLOGICAL AND PSYCHOLOGICAL PHENOMENA OF DREAMS AND APPARITIONS.

[No. III. of a Series.]

*Continued from Vol. IX. page* 561.

We would now direct attention to those powerful reminiscences in dreams, when past impressions which had been long forgotten come back like voices of departed spirits, unseen and unbidden. And probably there is not any class of phenomena which possesses more psychological importance in elucidating the science of Mind.

When we dream of events long since passed away, we seem to resuscitate all the persons necessarily connected with them, sometimes with the partial consciousness that these very persons have long since been dead. On other occasions we are at places and in countries we had never previously visited, and yet we give to the buildings a classic correctness, and preserve a distinct difference in the costumes of the inhabitants. In some instances we may recollect, on waking, that our knowledge of all these particulars we had derived from books, whilst in others we cannot remember any authority from whom we had received information. But in the dream, under all conditions and circumstances, there is no misgiving as to the actuality of all our impressions. These impressions of persons and things sometimes pass before the mental vision, not like dissolving views, but with a lifelike distinctness.

We may remark, on this interesting subject, that, when there is often in the waking-state a train of thought suggested by a word, a sound, a scent, &c., yet these involuntary efforts of the memory may reproduce on these impressions but merely faint outlines, just sufficient to renew the association and then rapidly pass away, and are shortly quite evanescent. But there is this marked difference in dreams—that, whilst there is reproduced persons and things which have been but superficially noticed in their actual conditions and existences, will become, during the vision of sleep, so vivid, as if they had delineated on the brain by a pencil of fire.

There are many other phenomena worth a brief notice. For instance, the working of the brain in some dreamers is not unlike that in certain forms of cerebral disease. The reminiscences seem to be hurried on helter-skelter, as in some species of delirium. Events, with all the *dramatis personæ* which are seen, float like indistinct *spectra*, and pass away before the sleeper seems to have had time to fully recognise them.

We may also mention, for the reflection of the psychologist, that sometimes, however, there are events which occur, and persons which are seen by the dreamer to him seem strange, neither of which does he recognise ; and even his waking intellect arrives at the conclusion that they have been the pure creations of the dream, so oblivious is the individual of any pre-knowledge of either the things or persons : but suddenly, however, there is a conviction of the verity of the events and actual existence of the persons seen in this vision of the sleep. And, curious enough, that such persons, although dead, "yet speak" with all their respective peculiarities during this mental resurrection of the dream. It has already been remarked, that in dreams of most kinds, the dreamer is himself the principal actor. This is a necessary consequence of his *egoism*, which gives him not only a personal identity, but tends, by preserving this intuitive perception of his own individuality, to furnish the best evidence of his psychological attributes. Hence, whether the mind reproduces past impressions or those of recent occurrence, they furnish an indubitable proof of its magical power over time and space. The mind, in fact, seems to be endowed with a Promethean daring, drawing fire from heaven, and has thus communicated life and energy to its own creations ; and thus its apparent eccentricities become one kind of evidence for its own immortality. All these phenomena may be quite compatible with perfect mental health. There are some persons who are known in their waking-state to exaggerate, even under the most ordinary circumstances ; it is, therefore, not any wonder that in their dreams they should be guilty of the greatest absurdities and of utter impossibilities during their sleeping adventures,—so that their dreams resemble some forms of insanity.* Thus, in the cases of which we speak, there seems to be not any *distinct* individuality. For a little, weak, vain man, even should he be emaciated, will in his dream conquer giants, and destroy with perfect ease the most ferocious animals, and this without the slightest misgiving as to the reality of the acts, or anything like doubt as to his own herculean powers.

An interesting illustration was related to us, which will explain the latter statements, even should a commission of lunacy pronounce the individual *non compos mentis*. The narrator and hero of the dream was a very small man, of a nervo-bilious temperament, very thin and very irritable. He said—"A short time before I got up, I had a dream, in which I saw that burly, thick-headed, Dr. A—— come to see us, accompanied by his tall,

* It is not our intention to discuss this subject, and we may merely remark, that a writer in a recent number of the "Psychological Journal" endeavours to prove a general resemblance between dreaming and insanity. We only contend for this special instance.

fat, eldest daughter. The latter asked, with her usual volubility, if we had heard some *new* compositions, with the names of the authors she enumerated, ending with *rini* or celli, and so forth. Before I could reply, her impudent father burst forth in a horse-laugh, saying, 'What does he know of music? Men of his age have not much appreciation of classical compositions. All these sort of folks can comprehend is some pretty little simple English ballad! Ha-ha-ha! what does our little friend know or care for brilliant and elaborate Italian music?' And he ha-ha!'d again and again. This attack aroused my *self-esteem*, and as I threw my head back, my stature increased amazingly, and my muscles acquired, instantaneously, a vast intensity of power; so, turning savagely on the burly doctor, I called him an impertinent fellow, and spun him round like a peg-top. He turned quite pale, and with a gruffish tone, and a bravado shake of the head, said, 'Pray don't do that again!' I was mightily pleased to think this great bullying boaster was somewhat cowed; then it was my turn to banter—'Well, Mr. Doctor,' I said, sarcastically, 'what are the fanciful and showy compositions of your modern *artistes*, to be compared to the Ambrosian and Gregorian chants, which, being somewhat altered from their original Hebrew compositions, will remain, from their inherent excellence, admired for their beauty and grandeur whilst the human mind can appreciate what is excellent and perfect in musical harmony?' In my vehemence on a subject of which I knew very little, as is usual in such dreams, I awoke, and then heard one of my own children playing a fantasia on the pianoforte, which no doubt had suggested the subject of the dream and the dispute, which, strange to say, was sustained with all the peculiarities of each speaker." The dreamer laughed at the absurdity of his moving such a huge mass as the doctor, and said, "It is, indeed, well that this adventure had occurred in a sleeping vision, for I am quite sure that had I in reality have attempted to do him any indignity, my burly friend would have despatched me with one blow."

We recollect meeting with a patient at a private asylum, who was a pigmy in stature, and as feeble as a boy, yet, when his *self-esteem* was offended, he would have a paroxysm of passion, and give expression to the most fearful execrations, with threats that he would crush the contemptible fellow who had so grossly insulted him. Once started, all seemed clear before him. Then he would continue for hours, and sometimes for days, to tell the most marvellous tales of his prowess, and the number of victims he had immolated to his ire; whilst his almost superhuman acts of strength he had performed, must have convinced all how dangerous it was to begin with him. And woe unto him who had the temerity to treat him with contumely,—death was not sufficient; he could only be satisfied by annihilating such evil-doers from out of all human society.

But to proceed—we may observe, that the individuality of the dreamer is even shown by the activity of some of the predominant powers of his mind. We do not mean to repeat a previously-stated view, "that in dreams of most kinds the dreamer is the principal performer;" this is a necessary consequence of the constitution of the mind. Nor is it simply because he fashions and changes past and present events that this individuality is rendered obvious. It is in the fact, that whatever may be the temper, disposition, and intelligence of the individual, he will manifest similar traits during sleep and when awake. Probably, in the latter state he might modify a statement, or remain silent when it might injure him; or he might permit himself to be literally "all things to all men," but in the dream; then his actual character shows itself; the exceptions we shall subsequently notice. If a man is irritable in his normal state, he will be highly choleric in the dream. If parsimonious naturally, his sleeping thoughts will be of gold, and so forth. Whilst in general, world-sensual men will have animal dreams, and moral men the reverse. Yet even these states are modified by the state of the health and the previous active or inactive state of the mental faculties in general. And we shall subsequently explain, that wicked, and even brutal dreams may be experienced by the moral and the most benevolent.*

In our experience, we have come to the conclusion, that dreams are often capable of furnishing a *moral* as forcibly as some of the ancient apologues. We will cite one example: A friend of ours happened to mention a curious incident, on a point of natural history, in a discussion on physiological subjects; and on the following evening whilst in the reading-room belonging to the society, he overheard, quite unintentionally, a small-minded man speak of his statement to another person, as "a traveller's tale," and as he immediately recognised the narrator, he looked silly and confused. Our friend, indignant at the implied charge of invention, simply addressed the libeller, saying, "Sir, although men of ordinary capacity, and extensive readers, recognize that though the incident mentioned is rare, it is not a solitary instance. But if there are in this institution weak persons who deny a fact, because it forms no part of their own experience, I'll obtain the evidence of the correctness of my report, from the gentleman who is in the possession of the speci-

* We shall have a curious physiological fact to expound in connexion with this topic. As there is a practical advantage to be deduced from these statements, we hope especial attention may be given to them.

men to which allusion was made, and which you have unfairly doubted." The small man made some shuffling apology, and for the time the affair dropped; but our friend, in a letter, repeated his statement, and received a reply endorsing its accuracy. He therefore took the first opportunity of reading the correspondence to the assembly in which he had previously narrated the fact which had been disputed. Still, he felt annoyed that anyone should have had the bad feeling to attribute to him the meanness of uttering an untruth, merely to excite the wonder of any assembly. The circumstances still irritated him, even after he went to bed, and which seem to have suggested a curious and singular dream;—he related it thus: "I thought that, somehow or other, I discovered great havoc among my papers, and found out that it was a mouse, deliberately, and with *malice prepense*, who had been the delinquent: there seemed to be great wantonness, as some of my rarest manuscripts were the most mutilated. And, as in dreams we are not very particular as to evidence, there seemed a perfect conviction that the only motive which induced the injury arose from a certain satisfaction which this creature derived from witnessing my anger and chagrin, as I had from time to time to remove the fragments. Every means to detect this secret enemy was, for a time, of no avail, and at length a mere accident revealed the mischievous little cowardly fellow, who every now and then stealthily came from his hiding-place, and slowly and deliberately commenced his depredations. But the moment his small, bright twinkling eyes caught a sight of me, he hurriedly sneaked away. This apparent impunity induced him to return again to his spiteful work, and as in my indignation I threatened him, he darted into the room, and rushed rapidly through an open window, and, although I commenced instant pursuit, I only succeeded in giving him a tap with a stick, at which he squeaked, and succeeded in escaping in safety. Some one then addressed me, saying—'It is well you did not kill it: if you had, there would have been a combination of some kind, to be revenged by the entire destruction of your life's labours.' This information made me doubly indignant, and, in the irritation of my mind, I awoke, and for a time I marvelled what could have suggested such a dream, and was much amused to think that it must have resulted from reflecting on the injury which such small minds could do, as he who had nibbled at my character, by designating an important physiological fact 'a traveller's tale;' and that he had not manifested any manly courage to dispute it openly, but had had recourse to a sly and secret method of injury. What petty jealousy! And was it sufficient punishment for his bad faith that I had justified the correctness of my communication, without fixing the blame of misrepresentation by naming the libeller? And yet I had a presentiment that if I had done so, such a contemptible nature would have tried other methods to disfigure my character. These thoughts have evidently induced the train of ideas which had taken the particular views of my sleeping vision. And hence it seemed true, that often there is a moral in a dream."

We must now trace some curious phases of dreams which result from partial fatigue of the brain; and when there is a state of complete sleep of the organs of the intellectual faculties and moral attributes, and an undue activity of the animal propensities. We admit that it is true in some measure, that dreams are modified by the pursuits of men, and by the natural character of individuals, although the latter is not always the case. And hence it will answer one of the objects of this essay, to explain an important physiological fact, and one which is too important to be passed by unnoticed, but which must not be regarded as counteracting any of our preceding views;—it may be considered as an exception:—it is, however, valuable for not confounding the accidental thoughts of a dream, as indicating the moral condition of men; and it furnishes important data to prove that pious and worthy people would do well to study the mental laws, for some of the best persons may dream of things and actions which disgust their waking consciousness, and in their sleep they may perform actions which may excite a sense of horror that, under any circumstances, they could entertain the most horrible conceptions.

The solution of this enigma is simple and easy, and so far as inductive reasoning is to be depended on, almost as satisfactory as if it could be actually demonstrated. When persons have been much engaged during the whole day on subjects which require the continued exercise of the intellectual and moral attributes, they may induce so much fatigue and exhaustion of these powers, that in their sleep, to their subsequent sorrow and surprise, they may have the most sensual and most vicious dreams! And they may, on awakening, under the violence of their own impressions, marvel at the fact that such should have been the case, as they had, even though tired, addressed the Lord with prayers for his manifold mercies and sustaining power, as manifested during their respective occupations. And they may perfectly recollect, that, prior to closing their eyes in sleep, they had not entertained any impure associations, or had occupied their thoughts with any reflections on the criminal condition of different members of the community.

On the contrary, they may remember that they had calmly looked back on a well-spent day, and, strongly impressed with one idea, that they might be permitted to continue their labours of usefulness with a renovated mind and body on the ensuing morning. And yet, under such a state of mind, and with such inclinations, nevertheless, scenes of the most polluted kind, and actions the most depraved, haunt the dreamy thoughts. Now, all this occurs from a non-observance of the Creator's laws. Such persons have sustained an undue exercise of the intellectual and moral powers, so as to induce intense fatigue in them, whilst the animal propensities had remained in a state of comparative inactivity. These latter powers (the animal propensities), being wide awake, they, in their turn, manifest greater energy in consequence, whilst the intellect revives imperfect impressions. Acting with the animal propensities, these revel as if under their respective *stimuli*, and hence the debased and brutalized dreams which result.

Had the intellectual attributes been more active and vigorous, they would have restrained in some degree, the vagrant thoughts; but, from their being in a dormant condition, they ceased to act as "the board of control," and therefore the propensities indicate the greatest excess;—memory furnishing a variety of incidents, either read or spoken of, whilst fancy aids in fanning the overstrained energy of the feelings, manifest their blind and impulsive condition, as when acting from the stimulation of real occurrences. These explanations furnish satisfactory data to explain the whole phenomena.

In saying these things we may still admire the wisdom of Zeno, which induced him to regard a dream as a test of virtue. For he thought (according to Plutarch) that if in a dream a man's heart does not recoil from vicious suggestions, there was an immediate necessity for self-examination and repentance.

If our explanation is correct—and numerous facts could be cited to render this obvious,—we demur at anything like actual responsibility for any thoughts which may actively occur in our dreams under the circumstances indicated. Even should our normal mental condition be shocked or disgusted that any such notions should have been entertained with all the vividness of reality, and though we repudiate any criminal responsibility from these visions of sleep, yet we, nevertheless, think that such dreams should be heeded. And if they occur involuntarily, we should begin to ascertain the cause, and endeavour to regulate in a better way both our mental faculties and the functions of the body. We should study the profound laws of God, by which we should ensure corporeal health and sanity of mind. We should recognise the importance of being "temperate in all things," and therefore avoid giving way to excess, even in sustaining the most elevated and spiritual thoughts; so that the exercise would then be natural, and in every instance invigorating and beneficial; for it is an indisputable truth, that when we regulate our varied powers, without excess of any kind, we ensure refreshing sleep, undisturbed by dreamy visions of any kind; and our lives would then be useful to others, agreeable to ourselves, and ensure a lasting satisfaction.

If the position is cramped, it may of itself be the source and the predisposing cause of a painful dream and a state of lassitude. But if there is an easy and natural position, and the bodily health is good, every shadowy reminiscence will be agreeable, and the individual will awake with a sense of being refreshed. We could cite many painful dreams which result from *dyspepsia*, in which the mind conjures up the most gloomy scenes and witnesses events of the most painful and harrowing kind; and it is equally tenable that lying on the back, by heating the *cerebellum*, will also be a cause of prurient associations, from the afflux of blood to that organ.

Imperfect as this essay must be considered, yet we deem a brief recapitulation of the arguments and explanations of dreams essential, prior to directing any special attention to apparitions; and our review will have another object—it will enable us to connect the disjointed portions, by showing that they still have some actual connexion.

In the first instance, we considered Nature's object in causing that complete state of rest of mind, and that passive state of the body, in sound sleep; we submitted evidence that the mental organs, like those of volition, suffered a condition of fatigue in the ratio of their respective exercise; that when there is sustained a certain amount of activity, so as to induce depression of the mental energies, and bodily lassitude, serious injury would result to the organic instruments, if some provision had not been made for their preservation;—that this renovation is effected in the act of sound and refreshing sleep; the various parts of the mental machinery of the bodily apparatus have lost portions of their materials by the frictional wear and tear resulting from excessive activity; and besides what they have sustained, they have lost some of the nervous power essential for their different functions; that this loss is renewed during perfect sleep; the *blood* supplies the new material for the purpose, and the nerves, those skilful workmen, apply this for repairing the machine. But for these salutary results, the brain must impart an impetus to keep the vital motions; and unless the brain is refreshed from its own labour, these results could not be effected. It is therefore essential for insuring these processes that the sleep should be sound, natural, and easy; for just as it is well known that unnutritious blood is poisonous and unfit for the process of reparation, so also the nervous fluid is vitiated when elaborated by an overtaxed brain; and then its tendency is, to induce a series of morbid impressions, and of disturbance of all the vegetative functions; and thus, conjointly, they become destructive to the happiness of the individual.

In course of these investigations we have clearly indicated four states of the muscular system, either as predisposing the kind of dream, or as directly inducing the phenomena during the sleeping vision:—

Firstly. The incapacity of moving, arising from some uncomfortable position of the limbs, inducing cramp; or from pressure on the heart, from lying on the left side, as in nightmare; in both phases affecting *per se* the circulation.

Secondly. When indigestion causes horrid dreams from exciting sensations of terror. Then the muscles seem partially paralysed, in the same way, and with similar consequences, as when a person in the waking-state is under the influence of strong fear.

Thirdly. When dreaming of some muscular effort being made, and continued for some time, which produces a similar exhaustion of nervous power, in the same way, and in a similar degree, as if a corresponding effort had been made in a state of complete consciousness, and with the perfect volition of the person.

Fourthly. When after a long illness there results a perfect prostration of strength, arising from extreme muscular debility, there will be disturbed dreams, in which are experienced great weariness and a sense of perfect incapacity of any manipulative power, and a sense of loss of locomotive power. In such cases the sufferer is invariably the victim of arbitrary and harsh treatment.

It has also been shown that dreams are ordinarily the result of partial rest of some of the cerebral organs and the over-activity of others; and that the congruity or incongruity of these nightly visions will depend on the number of the associated organs of perception, or their accidental defective association. That in the latter case ideas of the past are mixed up and stimulated by incidental sources of irritation from remote organs, when there exists a state of imperfect consciousness.

Whatever may be the subject of dreams, it will be confessed that they form an interesting episode of the nervous life, which is not only important to the physiologist, in tracing the connexion of such dreams to certain normal or abnormal actions of the living organism, but that they form a subject of interest also to the psychologist, as furnishing the latter with triumphant natural evidence that man's undying mind, however bound and fettered by its natural organization, seems to manifest greater power in proportion as the direct influence of the latter is partially suspended.

We are also taught a lesson in moral science from dreams. For, although we reject the inference of Zeno that such nightly visions are the reflex consequences of man's moral condition, yet we think one purpose would be answered by admitting his views, with certain qualifications, such as we have already indicated. We might, for example, apply, practically, some part of his recommendation,—that is, exert ourselves in the exercise of our moral perceptions, and thus acquire a great *surveillance* over our waking thoughts. Then, whenever in our nightly visions we become strongly affected with grossly prurient or cruel ideas, so diametrically opposed to our ordinary thinking, and which we had never entertained during our perfect and normal consciousness, we should then regard such dreams as indicating some form of nervous disturbance, and seek the aid of a physician to restore the organs implicated. For the importance and verity of these views, we may remark, that often the most morbid trains of thought result, both in our waking and sleeping state, from deranged secretions.

Lastly, we venture to assert, that the statements in this essay furnish positive data for explaining "the philosophy of dreams;" or, in other words, the different phenomena are confirmed on the *theory* of the predisposing causes which produce them.

It is often said that the whole period of a man's life is a dream. Such may be the case, figuratively speaking; but we should remember, that even in this life's dream there are vast distinctions and differences.

Much of the happiness of individuals depends on the proper exercise of the bodily and mental faculties. Hence there is, in some degree, more in the kind of education given than in any connate peculiarities. That if an individual is trained to obey the laws of health in reference to the bodily functions, and to regulate his animal appetites by the dictates of an elevated moral code, under the influence of a cultivated intellect; that he would be enabled by such an education to render the *feelings* the servants of his will, and, not as is generally the case, his imperious dictators. When such a mental training shall be given, then "the life's dream" will not only be more agreeable and satisfactory, but what is still more important, such a being may reasonably calculate to awaken from the sleep of death with powers more elevated and ennobled, and experience a conscious bliss without any disturbing influence, from a conviction that he has attained a *real* state of existence, in which all manifests the beautiful and the true, and where animal appetites cease to affect him.

It was from ideas of this kind which first suggested, and subsequently confirmed, the actual verity of apparitions. What, for example, more natural than to suppose our departed friends might visit the spots once dear to them, and in their spiritual capacity breathe a blessing on us? or, that they watched over our welfare with an additional solicitude, from their own experience of the various temptations which may warp the best disposed? or, that those who had trampled on the moral laws, which, by excluding them from scenes of bliss, had, as a consequence, doomed them to wander on the earth, and seek in the hours of night (so kindred with their own sullied natures), the very spots where their crimes had been committed, and which was to be to them an endless source of torture?

Apparition-seeing, then, differs from dreaming, in this particular,—that in the latter state we acquire an actual experience that all we have perceived in these nocturnal visions had not any actual existence, but were merely reminiscences formed by the active mind during our sleep; whilst, apparitions being seen when our senses can take cognizance of objects, and when they are apparently affected, normally, by their respective *stimuli*, it is not a matter of surprise that their *actual* existence should be admitted. These phenomena we shall, however, prove to be waking dreams!

Premising that, as a certain amount of exercise is essential to the health of the body and the mind, it is also true that over-exercise is fatal to both conditions.

In reference to the corporeal functions, when they are subject to excessive and too long-sustained fatigue, there is induced a state of lassitude which may end in such a degree of exhaustion as to merge on disease; and that if the mental faculties are so over-taxed as to induce a too susceptible condition, sufficient to banish sleep, many morbid ideas may be experienced, having reference to the previous state of the individual, his opinions, occupation, and so forth.

We may also incidentally remark, that when the perceptive faculties, in their normal states, are powerfully impressed with real objects, ideas or pictures of them are mentally perceived; and these impressions are retained, even when the objects which first produced them are no longer cognised. It matters not whether we recal them by the laws of association in the course of conversation, or whether they are revived in a dream; they have in both instances all the vividness of reality. There is, however, this marked difference, that in a normal state of mind there is a distinction made between the abstract ideas and the objects which suggested them. Thus, if we have seen certain persons, or visited particular localities, we have the power to reproduce either when absent from us. In the waking-state we call this memory, and during sleep we designate these revived impressions dreams.

In the latter, however, the continuity of the ideas may be disjointed, and our visions may be an heterogeneous combination; but so soon as we recover our perfect consciousness we distinguish what is incongruous and discrepant with our original experience.

To render our notion of what is the condition of the brain when apparitions are seen, we have only to notice the phenomena in *delirium*,—in which state all that passes in the fevered brain is regarded by the individual as real; and, in his case, there is an incapacity, as in ordinary dreams, to correct the complex and confused impressions which, to him, have an actual existence. We have witnessed many painful and amusing instances as confirmatory to the statements.

Thus, the visions of dreams, and in the delirium of fever, as being dependent on certain conditions of the brain, may be rendered important for elucidating the present section of our essay, particularly when we observe in connexion with this information the morbid trains of thought resulting from the depressing passions of *fear*, anxiety, despondency, and so forth.

If, for example, a person has been long indisposed, and he is of a naturally timid and cautious disposition, should he be left alone late at night, and in a solitary and quiet locality, it might prove fatal to his sanity. For every sound, under such circumstances, may induce emotions of fear, until the heart's action is greatly disturbed; and, with this disturbance, the imagination may be rendered morbidly sensitive, and then it is probable he may fancy himself addressed by name. And if this state continues for any length of time, he will people every nook and corner with dark, scowling faces, until so great may be his terror at these ideal personages as to produce fits, or delirium, or insanity; and then he is confirmed in his notion, that the spectrii have indeed a real existence.

In every vision, therefore, whether seen in dreams or delirium, or under the influence of narcotic poisons, or from the depressing passions, the perceptions are more or less implicated—in some measure confused and distorted. Thus, objects are either smaller or larger than they are in nature; or they present mere shadowy outlines, and are either very symmetrical, or distorted —and they may be regarded as realities, or otherwise. This will depend on the degree in which the sense of *marvellousness* is experienced, as it is this faculty which gives a tendency to seek for the supernatural, and derives a kind of excitement amounting to a morbid craving, for tales of wonder.

Akenside, in his "Pleasures of the Imagination," has recognised its influence, and the strong desire it induces for everything that can minister to its gratification, and concludes, with us, that it is essential for a belief in apparitions. He graphically tells us :—

"——————————— by night
The village matron, round the blazing hearth,
Suspends her infant audience with her tales—
Breathing astonishment!—of witching rhymes
And evil spirits; of the death-bed call
Of him who robbed the widow, and devoured
The orphan's portion; of unquiet souls
Ris'n from the grave to ease the heavy guilt
Of deeds in life conceal'd; of shapes that walk
At dead of night, and clank their chains, and wave
The torch of hell around the murd'rer's bed.
At every solemn pause the crowd recoil,
Gazing each other speechless, and congeal'd
With shiv'ring sighs: till, eager for th' event,
Around the beldame, all arrest, they hang,
Each trembling heart with grateful terrors quell'd."

And it is as obvious that the influence of this sentiment is experienced long after such had left the nursery. We therefore assert that, whatever may be the kind of spectre, it is seen by the mind's eye, and that it will be real, or the contrary, just as the *seer* possesses a strong sense of the marvellous, or otherwise. That this mental vision did not escape the penetrating intellect of Shakspear, we may cite the scene where the guilty Macbeth, under the combined influence of ambition, terror, and a strong marvellous tendency, was haunted by the instrument by which his intended murder was to be perpetrated. The celebrated dagger scene in Macbeth is true to Nature. Fevered by the *embryo* deed, and having been instigated to it by the satire and heroism of his wife, who had stirred up "his courage to the sticking point," we find him proceeding to effect the guilty act; and, although painfully awake, yet he dreams! Macready has comprehended the true philosophy of this extraordinary scene. He used to stand gazing on the outward space, with stormy and excited eyes, and in a tremulous, half-affrighted voice, begins—

"Is this a dagger which I see before me—
The handle toward my hand? Come, let me clutch
Thee.———"

To show that this fatal instrument was only seen as a consequence of his delirium, he continues—

"I have thee not, and yet I see thee still.
*Art thou not, fatal vision, sensible*
*To feeling as to sight?* Or art thou but
A dagger of the mind—a false creation,
Proceeding from the heat-oppressed brain?"

The whole soliloquy is a most extraordinary delineation of the effect of intense ideas on a susceptible organization, with an active temperament.

We may therefore understand, that if the mind of any one so conditioned is impressed with the recollection of any person who may have departed this world in the ordinary course of disease, or by a violent death, that the idea, or picture, may become so mentally vivid as to be seen as clearly as if the *original* had still existed in this matter-of-fact world.

The *how* and the *why* may be difficult to explain. If an object is placed before the eye, it is said to be painted on the retina, or *disc*, from the pencils of rays of light, which affects the optic-nerve. The impression is then conveyed to the brain, and there its actuality is appreciated by our consciousness; and this impression can be revived at any subsequent event or word which recals it by the association of ideas. Often these recollections are involuntary, as we may suddenly think of a friend, and, co-existing with the thought, is reproduced a mental picture of his individuality.

The state of mind called *reverie* but illustrates the condition essential to the seeing of apparitions. We met a friend, the Rev. U— W—, at a watering-place in the North of England. We had not met for years, and then it was at the hotel at which we were temporarily located. It was at the supper-table, and though mutually gratified, and undesirous of the meal, we did not leave the table, but sat unconscious of the presence of the strangers. We had an animated conversation on poetry, the drama, et cetera. Except preserving our respective personal identity, we were oblivious of our auditory, until a hearty laugh awoke us to our actual position. It was then that I remembered an engagement, but promised to return shortly; when I did get back it was late, having been detained longer than I had anticipated, the room was deserted, and the lights all extinguished except two, leaving "darkness visible," and in the imperfect glimmer I perceived my Reverend friend remained as motionless as a piece of sculptured marble! Was he asleep, or in one of his fits of abstraction? I spoke to him, but he answered not,—yet his full black eyes looked more than usually brilliant. I repeated my apology, as it was near "witching hour," but he neither heard nor heeded it.

Availing myself of the opportunity of studying the phases of this rare occurrence, I watched his humour. There was a change every now and then came over his feature, like fitful clouds, and yet so well delineated that one might speculate on the thoughts which so deeply interested him. At one time he appeared to be listening to exquisite music, for his head assumed the position of one who was deeply interested, yet, at the same time, judging of the degrees of merit of the composition. Then his features assumed the expression of one engaged in an animated conversation. It was evident that whatever might be the subjects or objects which affected him, were within his own capacious head. He did not distract his associations with any thing or object, around, above, or beneath him!

After permitting my friend to continue in this abstracted condition for an hour, I touched his shoulder with just sufficient force as might have awoke a sleeper. He stared as one just roused from a dream, when I told him why I had been detained, and that I only regretted it on my own account, as he had plenty of company during my absence. This ambiguous sentence left it an open question, whether or not he would allude to his reverie. His answer comprehends the whole philosophy of the phenomena we are investigating.

He said, "I did not mind the company you left me with, or recognise the time they themselves departed, because, Sir, I had better associates, companions, the honoured of past ages and the most worthy of the present. What interest do you think I could feel in the fat, purse-proud, fashionably dressed-up vulgarities, who were, in all probability, more satisfied with contemplating their own insignificant individualities, than they would have been with my conversation. Sir, I repeat, I have had companions of my own selecting—the best specimens of humanity in the olden times. I have conversed with them, heard their opinions enunciated in harmonious numbers, and been delighted to drink in the full streams of wisdom which they poured forth, not in any niggard manner. Nor did this seem a mere abstraction—a purely spiritual reminiscence; for to me they had all the reality of actual existence. I saw, heard, and to my inner sense, might have felt them."

Nor is this a solitary instance, when objects and persons in the outer world are unheeded, whilst the mind's eye recognises the creations of the ideality as real existences. This, then, is to all intents and purposes, spectre-seeing. The celebrated Baron von Swedenburgh, we are told, had constantly these waking-visions. It is related of him, that he would suddenly stop when walking in the street, and no one was near, yet he would make a low and most respectful bow, and continue his course. If interrogated why he had shown such a sign of reverence, he would say that "the Angel Michael, or Angel Gabriel, or some other spiritual being, as the case might be, had recognised him, when passing by him," &c.

It is impossible to regard such acts, by such a man, as the result of weakness of mind, or imbecility, as the Baron's philosophical works, and his able essays on natural history and natural philosophy, prove him to have been a man capable of manifesting the most profound thinking powers.

The true solution is to be found in his cerebral organization; for he had a capacious forehead, and a most extreme development of the faculty of *marvellousness.* He, though capable of great intellectual efforts, had a tendency to *spiritualize* material objects, and *materialize* spiritual entities.

We may also cite a more recent instance—that of Blake, the artist, well known as having illustrated "Blair's Grave" and "Young's Night Thoughts." It is said of him that he was frequently found in his studio, painting away with great energy, and with an expression of profound reverence, fixing his eye on a vacant part of the room, as if he was minutely studying some aristocratic sitter. If he observed an intruder enter he would silently motion him to depart, and afterwards make an apology for his seeming rudeness, probably telling his friend that he was painting Moses, or Abraham, or some other worthy of antiquity. We ask what were these *ideal* sitters but actual apparitions—the beings of his vivid ideality, revived by strong and vivid reminiscences of works in which they had been delineated, and which he had reproduced in his waking-dream, and through the influence of his powerful organ of marvellousness he had regarded them as living personages?

Before we proceed to enter into the greater speciality of this part of our essay, we must offer a few remarks on *double* consciousness.*

There is a purpose and a final cause for the *duality* of the mental organs, as this may be—as in the case of the organs of external senses,—as a prevention of exhaustion from over-fatigue. Because when one hemisphere is tired the other hemisphere could perform the respective functions. In the same way as we may exercise one eye, or one ear, and then the other, Dr. Wigan, in his work on the *duality* of mind, has confounded an important and distinct subject; for we cannot confound the instruments with agents by which they are manifested. We admit his explanation of the metaphysical faculty of *attention,* and think with him, that this is a result of the simultaneous exercise of both hemispheres of the brain on one subject. That

* It will be remembered that we have already noticed the duality of the mental faculties, as resulting from two distinct hemispheres of the brain.

when both are attempted to be used for different kinds of thought, at one and the same time, the effect will be to enfeeble both. We merely now allude to the double set of mental organs, that we may explain the *double* consciousness, such as is carried on by the dreamer in his questions and answers to the persons of his sleeping reminiscences, and that of the apparition—relevations, in certain morbid conditions of the human mind.

How can we otherwise reconcile the fact that in dreams, spectre-seeing, delirium, and insanity, persons will hear voices which have not any existence but in their own brains. Such persons will hear themselves called, warned, reproved, and encouraged by the peculiar activity of one side of the brain (acting with the auditory and optic nerves), from its being in a different state to the opposite hemisphere.

We are told of a double consciousness, when one hemisphere of the brain is affected and the other normal,—that the one has insane ideas, which are corrected by healthy organs (see Gall's report of the case of Dr. Moser, of Vienna), when this actually occurred. We have often heard insane persons, from the same cause, go on sustaining a conversation for some time, when no one is speaking to them. They mistake the train of thought which occupies one hemisphere for some one addressing them, and they answer with the other.*

But this double consciousness may exist in healthier minds—as, for instance, in Lord Brougham and Cobbett. We saw the first write in the Court of Chancery, and attend to the pleading of Sir William Hone; whilst the other discoursed with us, and continued to write his "Register."

We mention these facts, as this pre-knowledge is essential to understand our subject; and we must bear in mind that the causes of double consciousness may be irregular circulation,—a want of uniformity in the action of *stimuli*, the greater activity induced in one hemisphere by the disease of fever, &c. Besides these well-observed conditions, we have the involuntary activity of some of its particular organs, either from their being previously much excited, or from some systematical and extreme cultivation. In either case inducing a fixed habit of thought; and the latter is often a cause of mental disease.

* Nor is this double consciousness confined to the dreamer or madman. Most persons of active and energetic minds are liable to be thus influenced. For example, a young man, full of energy, may have trains of thought dictated by his animal propensities, whilst their impetuosity and blind impulses as to consequences must be counteracted by a train of better motives suggested by the moral sentiments and the intellect. And if the latter have been duly cultivated, the protest of his better nature will often be heeded, and save the incipient offender from remorse or disgrace.

Some years since a circumstance occurred at Hull, which, though partaking of the marvellous, is capable of being explained on the above data, and we record it from having known the individual, who was an active member of the Mechanics' Institute. Mr. ——, a whitesmith, had a son associated with him in his trade. They were both exemplifications of the disadvantage of "a little learning," if we test them by their sceptical notions on the immortality of the soul. One day they had been drinking together, when the father and son entered into a solemn engagement, that in the case of the death of either the departed spirit should revisit the earth, and make his presence known to the survivor. The son, soon after this, sickened and died, and, in all probability, the old man had a misgiving that the loss of his son was a penalty of their presumption. And thus his mind was constantly disturbed by this contumaceous compact, and ultimately it induced a monomaniac condition. On one occasion, when the old man had been working very hard over his furnace on a very hot day, and had, in all probability, taken more beer than usual, he reports that, after a moderate supper, he went to bed, when, soon afterwards, he perceived his room filled with brilliant lights, and his sense of hearing was delighted with the sweetest and most plaintive music. He raised himself up under an ecstatic state of feeling, when he beheld a figure clothed in white approaching him; it smiled, looked kindly in his face, and as it disappeared, he recognised his deceased son! That he then awoke his wife, and in a very excited manner told her of the vision, when she said, "O don't think anything about it, you have been dreaming!" Yes, we believe it was a waking-dream. The intense light of the forge had acted on the retinæ, and the extra stimulation had affected his irritable brain, which, with his previously long-cherished anticipation of such a visitation, had induced the whole phenomena. He had, it would seem, mentally perceived some such revelation, and being naturally *marvellous*, the wish was father to the thought, and under some peculiar condition of the brain, the anticipated event was realised.

The first correct notions we acquired on spectre-seeing was from a paper published by the late estimable Dr. John Alderson, entitled "An Essay on Apparitions," in which these appearances are accounted for by causes wholly independent of preternatural agency. After an examination, in the introduction, of the universal belief in ghosts and visions, he concludes by referring such phenomena to disturbance of the corporeal functions. In other words, all his own examples are in cases of *delirium tremens*.

We may incidentally mention, that sudden shocks to the nervous system often induce lamentable consequences, particularly with females.

We will merely state one example. Mrs. R——, a highly nervous young woman, had been sitting up to watch her dying uncle, to whom she was greatly attached. Early in the morning, just after she had fallen into a doze, he died. Some person in the room at the time the event occurred awoke her, and suddenly told her what had happened; the consequence was, a most serious hysterical attack. Just before the coffin was screwed down, some few days after this latter occurrence, the same officious person induced poor Mrs. R—— to feel how cold her uncle felt; she did so, and then followed another frightful attack. It was three or four years after this, that we saw her under one of these fits, and certainly nothing could be more painful. There was a choking sensation, with a most unpleasant huskiness of the voice. This was followed by a tetanic condition of the muscles of the neck, chest, &c., and a complete engorgement of the blood-vessels; and what was most singular in her case was, with the appearance of one spectre-stricken, gazing in a vacant manner at some spectacle which she saw.* She gave a most piercing shriek, shivered, and then became so perfectly shut out from the outer world, that nothing could affect her senses or rouse her from her torpor; and the hand with which she had touched her dead uncle was as cold as a dead limb could be, whilst all the rest of the body was at a higher than the normal temperature, and we were told that whatever reminded her of her uncle, immediately reproduced the fit with its different painful phases.

We have often seen cases where spectre-seeing had been induced by the excessive use of narcotics, similar to those which are so common with great drunkards. One example will suffice. Mr. R——, a man of a superior order of intellect, but whose moral character was much debased, would, in the midst of a conversation, turn his head suddenly round, and chide some fancied intruder in language neither courteous nor refined. Being curious to know what his waking-dream was like, we asked his man-servant what could so disturb his master, and induce him to give way to such bursts of anger without any apparent cause? "Why, see, he fancies that an old crow is constantly pecking at his right shoulder; and it is terrible to see him after he has taken a more than usual dose of laudanum: then he turns round to this ghost of a crow in a most savage manner, and swears at him in a most vehement way."

In this case the erroneous impression was impressed so vividly

* She, on the occasions, saw her uncle—his funeral, and all that had previously developed her painful affection.

on his brain that, under every circumstance, it haunted his imagination, and it became chronic. The annoyance was aggravated to such a degree that he believed in the conviction of its being a crow with an extraordinary tenacity, without the slightest remission of the apparition. We have had confirmation of our previously-stated solution of the waking-dreams, and select the following, in which, although apparitions were constantly seen, the seer regarded them as optical illusions. We had occasion to visit H——, in Yorkshire, and called to see an old medical friend, who said, with a strong Yorkshire dialect, "I have a regular 'puzzler' for phrenologists—a man who sees ghosts, and don't believe in them." We expressed a wish to be introduced to him. The individual was a burly man, of the name of B——, with a large head, and strong perceptive faculties, which projected to such a degree as to render his eyes deeply seated. His temperament was nervo-sanguineous, and he had for years exercised the craft of a blacksmith. In a few minutes after our conversation commenced, it was evident that he was a natural sceptic in all things, besides ghost-seeing—a sort of village Spinoza. After asking him what kind of apparitions he saw, he replied, "Sometimes they are d—d ugly—then I send them adrift; but when they are very handsome, like the poor simpletons paint their saints, then I detain them, and describe them to my wife." "Do you see these spectres with your eyes shut or open?" we asked. "Why, it makes no difference; I see them either way." "Are you always in the dark when they make themselves visible?" "No; they come sometimes in the dark, and sometimes where there are lights." "Have you any pain or uncomfortable sensation about any part of your head at the time, or prior to your seeing them?" "Yes, sir; a sense of fulness between the eyes and over the eyebrows." In this case there is the clearest evidence that Mr. B——'s ghosts were purely mental, and they were the result of the involuntary activity of the perceptive faculties, like the visions in dreams. In Mr. B—— this condition may have been induced by his previous laborious occupation (for he had then retired from business), and from his habit of drinking quantities of beer. But then there was the "puzzler," he did not believe in what he saw; and when asked why he did not, he attempted a *rationale*, by saying, that he laboured "under an optical illusion!" This we refuted, as he saw these apparitions distinctly, whether his eyes were open or shut, in the dark and in the light. But on examining his head, the part considered by phrenologists as the organ of *marvellousness*, was so deficient, that it seemed as if the convolutions in that locality had been scooped out; so we concluded that without that wonder-quality men might see apparitions and not believe the evidence, apparently, of their own eyes; whilst on the contrary, should *spectra* be seen, with the marvellous-faculty, it mattered not what might be the disturbing cause, the actual existence of such apparitions would be entertained.

We have known cases of the "ghost-seeing" when wide awake, which have been cured by leeches at the front of the forehead, just over the eyebrows,—evidently indicating that they have resulted from a congested state of the perceptive faculties.

We will now conclude with a circumstance which occurred to ourselves, and which is a confirmation of the advantage of knowledge. We were on a visit at N——, in Nottinghamshire, and had dined with a most respectable surgeon, and had taken more wine than usual. It was in the summer time, and the weather very hot and dry, which combined circumstances rendered us feverish and uncomfortable. It was late when we returned to our lodgings, and our sleeping-room was very small and ill-ventilated. We went to bed, but not to sleep, and tossed and tumbled, changing our position every moment, but was too restless to repose; at length we turned towards the window, and perceived between it and the bed there stood a short, thickset, burly figure, with a huge head, staring us in the face. Certainly nothing could appear more real and substantial, and after gazing on this monstrous creature, we put out our hand, when the monster opened his ponderous jaws and bit at us. We tried various experiments with the creature,—such as putting our hand before his face, which seemed to cover part of it. The longer we contemplated it the more palpable was this figure, and the more wrathful were his features. Struck with the apparent reality of the apparition, we mechanically felt our pulse: it was throbbing at a fearful rate; our skin was hot and dry, and the temporal arteries were throbbing at a railway speed. This physical condition had produced the phantom. We then jumped out of bed, when the spectre seemed to be nearer, and of more gigantic proportions. We then threw open the window to admit a little air, sponged our head and body, and thus, by removing the cause, the monster disappeared. Was not this a waking-dream? and is not the fact a perfect demonstration that, in this instance, a knowledge of the cause was more than "half" the cure.

We may now remark, in conclusion, that however varied the phenomena which have been treated, it will be obvious that in dreams and in spectre-seeing there is invariably some disturbance of the material organization, by which the mind acts in this life; and that all the means which are used to lessen undue action on any of the organs of vegetative life, will produce similar advantages when applied to lessen any abnormal condition of the brain, unless there exists a lesion of this important instrument.

Thus we have shown that spectres or apparitions are similar to the visions in sleep; that in both kinds of phenomena there is an involuntary activity of the perceptive faculties; and that, therefore, though apparitions under such conditions are real objects, from exalted or diseased perceptions, they should not be regarded as objects of terror, but simply as unmistakable indications of some disturbed cerebral action, and that it would be wise in such cases to apply for medical advice.

Lastly, we may observe that in examination of dreams and of spectral illusions, we have restricted our investigations to such data as are within the range of physiological science. In doing so, we do not deny the truth of prophetical visions, but refer them to another kind of evidence, an evidence which it is not the province of a psychological journal to discuss, and, therefore, has not formed any part of this sketch of a complex but interesting inquiry.

---

